# Electronic cigarette use in Saudi Arabia: a cross-sectional study on emerging trends and public health concerns

**DOI:** 10.3389/fpubh.2025.1574623

**Published:** 2025-08-14

**Authors:** Farah Kais Alhomoud, Ayat Almuhayshi, Roqaia Altarouti, Tuqa Abushaheen, Faten Alhomoud, Noor Alotaibi, Dana Alsugeir, Khalid A. Alamer, Yousef Alqarni, Basmah Alfageh, Hailah Almoghirah, Fahad Alsulami

**Affiliations:** ^1^Department of Pharmacy Practice, College of Pharmacy, Imam Abdulrahman Bin Faisal University, Dammam, Saudi Arabia; ^2^Department of Clinical Pharmacy, College of Pharmacy, King Saud University, Riyadh, Saudi Arabia; ^3^Clinical Pharmacy Department, College of Pharmacy, Taif University, Taif, Saudi Arabia

**Keywords:** e-cigarettes, Saudi Arabia, smoking cessation, nicotine, public health policy

## Abstract

**Background:**

Electronic cigarette (e-cigarette) use has become increasingly popular worldwide, including in Saudi Arabia, especially among younger individuals. This study investigates the patterns, motivations, and perceptions of e-cigarette use to inform public health interventions and policy.

**Methods:**

This descriptive cross-sectional study surveyed traditional cigarette smokers and ex-smokers above 18 in Saudi Arabia using an online questionnaire distributed via social media with convenience and snowball sampling. The tool, adapted from validated sources and refined through expert review and pilot testing, captured demographic data, smoking behaviors, perceptions, motivations, and patterns of e-cigarette use. Closed-ended questions ensured consistency in responses. A power analysis was conducted to determine the required sample size. Descriptive statistics, chi-square tests, one-way ANOVA, correlation analyses, and multinomial logistic regression were applied (*p* ≤ 0.05).

**Results:**

Findings revealed that e-cigarette use was significantly higher among younger males (*p* = 0.028), with 86% of participants being male and 63% aged 18–28. Socioeconomic factors such as lower income and education levels were associated with higher usage rates (*p* = 0.001 and *p* = 0.030, respectively). Dual users of e-cigarettes and traditional cigarettes made up 47% of participants, while 31% were ex-smokers, and 22% were exclusive e-cigarette users. A preference for high nicotine content (≥16 mg) was noted among 26% of participants, and 44% reported adverse effects like shortness of breath and chest pain. Key motivations included smoking cessation (45%), flavor variety (35%), and perceived safety (29%). Retailers (43%) and online stores (27%) were the primary sources. Notably, 61% of participants supported government regulation of e-cigarettes. Gender, region, marital status, and education were significant predictors of usage patterns (*p* < 0.05).

**Conclusions:**

E-cigarette use is widespread among younger males in Saudi Arabia, driven by socioeconomic factors and perceptions of safety. Despite these perceptions, adverse effects like shortness of breath and chest pain were frequently reported. Comprehensive public health measures, including nicotine regulation, sales restrictions, taxation, and education, are urgently needed. Collaboration between the health and education sectors, along with continuous monitoring and research, is crucial for guiding effective national strategies.

## 1 Introduction

Electronic cigarettes (e-cigarettes), commonly referred to as vapes, are battery-powered devices that heat a liquid to produce an aerosol, often mischaracterized as “vapor.” This aerosol, a mixture of small particles, is inhaled by the user and can also be passively inhaled by bystanders when exhaled (1). The liquid typically contains nicotine, the addictive component of traditional cigarettes, and may also deliver other substances, such as cannabis or illicit drugs. E-cigarettes are frequently marketed in vibrant designs and flavors appealing to younger demographics, including fruit, candy, menthol, and mint. The act of using these devices is commonly referred to as “vaping” (2, 3).

Between 2020 and 2024, e-cigarette sales in the United States increased by 34.7%, reaching 21.1 million units (4). Disposable devices accounted for 58.1% of sales, while non-tobacco flavors, such as menthol and fruit, comprised 80.6%. During this period, e-cigarette revenue totaled $488.9 million (4, 5). In contrast, comparable data on e-cigarette sales trends in the Kingdom of Saudi Arabia (KSA) remains limited. However, the use of e-cigarettes has gained significant popularity in the country, particularly among young adults, likely driven by the availability of youth-oriented flavors and the increasing accessibility of these products (6). This trend aligns with global patterns, where the rapid expansion of e-cigarette marketing through online platforms and small retailers, combined with a diverse range of appealing flavors, has contributed significantly to their widespread adoption (3).

In response to the growing prevalence of e-cigarette use and its associated public health concerns, the KSA has implemented comprehensive regulatory measures. These include a 100% tax on tobacco products, such as cigarettes and water pipe tobacco, introduced in June 2017 by the Saudi Ministry of Health (MOH) and the Saudi Food and Drug Authority (SFDA) as part of a broader strategy to reduce tobacco consumption (7, 8). Additional efforts include prohibiting the sale of tobacco to individuals under 18, restricting smoking in public spaces, and imposing fines for violations. Furthermore, regulations have been introduced to limit the quantity of tobacco products allowed for personal import (9). While e-cigarettes fall under tobacco product regulations in Saudi Arabia, including taxation and import restrictions, the SFDA does not currently approve e-cigarettes as smoking cessation devices or endorse their safety (7, 8). This regulatory ambiguity highlights the need better to understand usage patterns, perceptions, and associated risks. While these measures represent a significant step toward curbing e-cigarette use, further research is needed to address the growing prevalence and comprehensively examine the knowledge, perceptions, and behaviors associated with e-cigarette use among diverse user groups in the KSA, including current users, former users, and individuals who use both traditional and electronic cigarettes. Furthermore, this study seeks to identify key factors driving e-cigarette use across various provinces in the Kingdom. The findings are intended to provide evidence-based insights to support the development and refinement of public health policies and targeted interventions to mitigate the risks associated with e-cigarette use.

## 2 Methods

### 2.1 Study design, setting, sampling, and data collection

This study utilized a descriptive, cross-sectional design with data collection conducted over 3 months (from October to January) after obtaining ethical approval from the Institutional Review Board. A questionnaire-based approach was employed to investigate e-cigarette-related behaviors and perceptions among smokers and ex-smokers aged 18 years and older residing in the KSA. The sample was obtained through a combination of convenience sampling and snowball sampling methods. The survey was disseminated via social media platforms to maximize reach and engagement. The use of online platforms emphasized recruiting young adults, as this demographic represents a significant proportion of e-cigarette users and could be effectively reached through online platforms. The survey was presented in English. The decision to administer the survey in English was based on several considerations:

- Target population proficiency: our study targeted smokers and ex-smokers, predominantly young adults and university-educated individuals (69% of participants had at least a university degree). This population is expected to have sufficient English proficiency, as English is the primary language of instruction in health-related programs and is widely used in higher education and professional settings in Saudi Arabia.
- Consistency of validated measures: the questionnaire items were adapted from validated tools and previous studies conducted in English. Administering the survey in English helped ensure consistency in technical terminology (particularly regarding e-cigarettes) and minimized the risk of misinterpretation during translation.
- Pilot testing for clarity: prior to launching the survey, it was pilot-tested with a sample of smokers. Feedback confirmed that participants comprehended and responded to the English questionnaire without difficulty.

The selection of an online survey method was particularly suitable given the prevalent online availability of e-cigarettes and the necessity of targeting users across diverse geographic regions. An introductory information page was included to provide potential participants with a comprehensive explanation of the study's purpose, objectives, and inclusion criteria. Participants were required to review this information carefully, after which they provided informed consent by electronically agreeing to participate through a click-wrap agreement embedded within the survey interface.

### 2.2 Study questionnaire

A structured questionnaire was developed through a comprehensive literature review (3, 10, 11), to ensure alignment with the study objectives and quality assessment domains. An advisory group of three pharmacy academic experts reviewed the initial draft to refine the content and wording for clarity and relevance. The finalized questionnaire consisted of two sections: demographic data and participants' behaviors, beliefs, and awareness regarding e-cigarettes, including factors influencing their use.

To validate the instrument, a pilot test involving 12 randomly selected smokers was conducted to assess clarity, ease of use, feasibility, and face validity. Feedback from the pilot was used to refine the questionnaire; however, data from the pilot were excluded from the final analysis. Closed-ended questions were used to streamline data collection and reduce bias.

Reliability was ensured by rigorously documenting the data collection process and adhering to the study protocol. Data were gathered on a single occasion to prevent variability and support reproducibility. These steps enhanced the instrument's validity and reliability, providing a robust foundation for capturing accurate and actionable data.

### 2.3 Sample size

A power analysis was performed to calculate the required sample size for the study, assuming an unknown population size. To achieve a statistical power of 80% and a 95% confidence interval, with a 5% margin of error and an anticipated 20% non-response rate, the estimated sample size was determined to be 420 participants. The calculation was based on a formula informed by data from a previously published study (12), ensuring methodological rigor and the reliability of the sample size estimation.


(1) $$\begin{array}{l} n=\;\frac{{\left(Z_{1-\beta }+Z_{\alpha /2}\right)}^{2}\left[p\left(1-p\right)\right]}{d^{2}} \end{array}$$

### 2.4 Data analysis

Data were analyzed using IBM SPSS Statistics software. Descriptive statistics summarized demographics, usage patterns, and reasons for e-cigarette use, with chi-square tests used to assess relationships between variables (*p* ≤ 0.05). One-way ANOVA compared practices, knowledge, and perceptions among dual users, ex-smokers, and exclusive e-cigarette users, identifying significant group differences and trends relevant to e-cigarette use in the KSA.

### 2.5 Ethical approval and considerations

Ethical approval for the study was secured from the Institutional Review Board (IRB) under Vide Letter No. IRB-2024-05-735 dated October 15, 2024. Informed consent was obtained from all participants, ensuring adherence to ethical standards and the protection of participant rights throughout the research process. In addition, this study was conducted in accordance with the principles of the Declaration of Helsinki.

## 3 Results

### 3.1 Participant demographics and socioeconomic characteristics

The study included 441 participants aged 18–72, with the majority being male (*n* = 379, 86%) and aged between 18 and 28 (*n* = 277, 63%). E-cigarette use was significantly more common among younger participants (*p* = 0.028), with male reporting higher usage rates than female (*p* = 0.001). Regarding smoking behavior, 47% (*n* = 209) of participants were dual users of traditional cigarettes and e-cigarettes, 31% (*n* = 134) were ex-smokers, and 22% (*n* = 98) exclusively used e-cigarettes.

Socioeconomic and demographic characteristics revealed that nearly half of the participants (*n* = 210, 48%) had a monthly income below $1,333. At the same time, e-cigarette use was significantly higher among individuals with lower educational attainment (*p* = 0.030) and lower income levels (*p* = 0.001). Most participants (*n* = 395, 90%) reported living with or having close contact with another e-cigarette user. The majority resided in the Eastern Province (*n* = 232, 53%), and most participants had a university education (*n* = 304, 69%; Table 1).

**Table 1:** Demographic characteristics of participants (*n* = 441)

**Variables**	**Numbers**	**Percentages**
**Age (years)**		
18–28	277	63
29–39	110	25
40–50	42	10
51–61	11	3
62–72	1	0.2
**Gender**		
Male	379	86
Female	62	14
**Marital status**		
Single	267	61
Married	163	37
Divorced	10	2
Widowed	1	0.2
**Ethnicity**		
Arabs	435	99
Non-Arabs	6	1
**Monthly income**		
-SR 5,000 (-$1,333)	210	48
SR 5,000-10,000 ($1,333-2,667)	103	23
SR 11,000-16,000 ($2,933-4,267)	66	15
SR 17,000-22,000 ($4,533-5,867)	31	7
>SR 22,000 (>$5,867)	31	7
**Region of residence**		
Eastern province	232	53
Middle province	107	24
Western province	73	17
Southern province	17	4
Northern province	12	3
**Education level**		
University	304	69
High school or less	114	26
Postgraduates	23	6
**Live with/friend of e-cigarette smoker**		
Yes	395	90
No	42	9
Don't know	4	1
**Type of smoking**		
Dual users (use both e-cigarette and traditional cigarette)	209	47
An ex-smoker who used e-cigarettes	134	31
E-cigarettes user who never smoked traditional cigarettes	98	22

### 3.2 E-cigarette use patterns and practices

Among the 441 participants, 43% (*n* = 188) had used e-cigarettes for < 1 year, 30% (*n* = 132) had quit, and 27% (*n* = 121) had used them for over a year. Daily usage was reported by 50% (*n* = 218) of participants, while 7% (*n* = 32) used e-cigarettes at least once a month. Regarding the timing of their first e-cigarette of the day, 27% (*n* = 118) smoked after 1 h of waking, and 19% (*n* = 84) within 6–30 min.

Nicotine-containing e-cigarettes were used by half of the participants (*n* = 219, 50%), with 26% (*n* = 113) preferring e-juices with ≥16 mg nicotine and 24% (*n* = 106) choosing < 9 mg. Nicotine-free flavored e-cigarettes were used by 18% (*n* = 81) of participants. Retailers (*n* = 188, 43%) and online stores (*n* = 119, 27%) were the primary sources of e-cigarettes. Significant differences were observed among dual users, ex-smokers, and exclusive e-cigarette users regarding the duration of e-cigarette use (*p* = 0.054), usage frequency (*p* = 0.046), and preferred types of e-juice (*p* = 0.001; Table 2).

**Table 2:** E-cigarette usage practices among participants (*n* = 441).

**Variables**	**Numbers (*n* = 441)**	**Percentages**	**Dual users of both (*n* = 209) (*p*-Value)**	**Ex-smokers (*n* = 134) (*p*-Value)**	**E-cigarettes smokers (*n* = 98) (*p*-Value)**
**Frequency of e-cigarette use**					
At least once a day	218	50	**0.046**		
At least once a week	40	9			
At least once a month	32	7			
Others	151	34			
**Use of e-cigarettes**					
Stopped smoking e-cigarette	132	30	0.054		
Over than a year	121	27			
Over than 6 months	86	19			
Over a month	55	13			
Less than a month	47	11			
**First e-cigarette after waking**					
After 60 min	118	27	0.128		
6–30 min	84	19			
5 min	53	12			
31–60 min	48	11			
Others	138	31			
**E-juice nicotine levels**					
≥16 mg nicotine	113	26			
≤ 9 mg nicotine	106	24			
Without nicotine with flavors E.g., mango, mint, Red Bull, grape, and vanilla	81	18	**0.001**		
≤ 12 mg nicotine	36	8			
Without nicotine (no flavors)	10	2			
Do not know	95	22			
**E-cigarette source**					
Retailer	188	43	0.974		
Online stores	119	27			
Exchange among peer	63	14			
Wholesale	47	11			
Both seller	17	4			
Family member	7	2			

p-Value was calculated using the independent samples t-test. Significance determined at p ≤ 0.05 (two-tailed). Bold values indicate statistically significant results (*p* < 0.05).

Correlation analyses were conducted to explore associations between e-cigarette use frequency, type of e-juice used, and intention to be a regular smoker in the following year. The results showed a weak but statistically significant positive correlation between e-cigarette use frequency and type of e-juice used (Pearson's *r* = 0.113, *p* = 0.006; Spearman's rho = 0.138, *p* = 0.001), and a moderate negative correlation between e-cigarette use frequency and intention to be a regular smoker (Pearson's *r* = −0.464, *p* < 0.001; Spearman's rho = −0.464, *p* < 0.001). To further identify independent predictors of e-cigarette use patterns while controlling for potential confounders, a multinomial logistic regression was performed with smoking group membership as the dependent variable (ex-smokers who use e-cigarettes, exclusive e-cigarette users who never smoked traditional cigarettes, and dual users). The analysis revealed that gender (*p* = 0.000), region (*p* = 0.000), marital status (*p* = 0.007), and education (*p* = 0.014) were significant predictors in distinguishing between these groups (*p* < 0.05).

### 3.3 Motivations for e-cigarette use

The primary motivations for continued e-cigarette use were smoking cessation (*n* = 197, 45%) and the availability of diverse flavors (*n* = 156, 35%; Table 3). Additionally, nearly half of the participants (*n* = 205, 47%) indicated a willingness to try flavored e-cigarettes, while 35% (*n* = 155) expressed an unwillingness to try unflavored variants. Participants perceived e-cigarettes as safer compared to traditional cigarettes, hookah, or shisha (137.29%; Table 3).

**Table 3:** Key factors influencing e-cigarette use among participants (*n* = 414).

**Factors**	**Numbers**	**Percentages**
To quit smoking	197	45
Flavor options (e.g., fruits)	156	35
For enjoyment	137	31
Easier than regular cigarettes	132	30
Perceived as safer	129	29
Curiosity	103	24
Peer influence	102	23
Cost-effectiveness	96	22
Stress relief	69	16
To cope with sadness	43	10

### 3.4 Perceptions and beliefs about e-cigarettes

**Figure 1:**
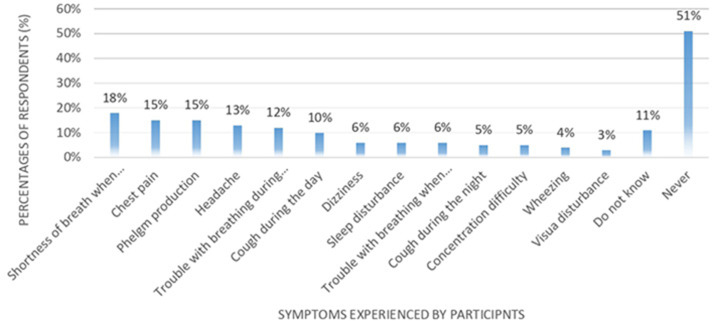
Primary sources of information about e-cigarettes.

Over two-thirds of participants (*n* = 306, 69%) reported learning about e-cigarettes through neighbors or friends, while the Internet (*n* = 162, 37%) and media (*n* = 137, 31%) were secondary sources (Figure 1).

Since participants could choose multiple sources, the total percentages exceeded 100%. When asked to define e-cigarettes, 89% (*n* = 393) described them as devices that produce a mist resembling smoke but without containing tobacco or nicotine. Conversely, 11% (*n* = 48) considered them similar to traditional cigarettes in function but differing in shape and perceived as safer. No significant differences were observed in definitions across user groups (*p* = 0.228).

Among the 441 participants, 61% (*n* = 269) supported government regulation of e-cigarettes, 56% (*n* = 247) believed they are less addictive than traditional cigarettes, and 55% (*n* = 243) felt they facilitate smoking cessation. Significant differences in perceptions were observed across user groups. Dual users were more likely than exclusive e-cigarette users or ex-smokers to view e-cigarettes as primarily used by smokers (*p* = 0.002), as posing health risks (*p* = 0.049), and as requiring regulation (*p* = 0.005; Table 4). These results underscore the influence of smoking behavior on perceptions of e-cigarette risks and regulatory needs.

**Table 4:** Perceptions of e-cigarette use among participants (*n* = 441).

**Smoking status**	**Dual users (*n* = 209)**	**Dual users (*n* = 209)**	**Dual users (*n* = 209)**	**Ex-smoker (*n* = 134)**	**Ex-smoker (*n* = 134)**	**Ex-smoker (*n* = 134)**	**E-cigarette smoker (*n* = 98)**	**E-cigarette smoker (*n* = 98)**	**E-cigarette smoker (*n* = 98)**	***p*-Value**
**Statements**	**Agree** ***n*** **(%)**	**Don't know** ***n*** **(%)**	**Disagree** ***n*** **(%)**	**Agree** ***n*** **(%)**	**Don't know** ***n*** **(%)**	**Disagree** ***n*** **(%)**	**Agree** ***n*** **(%)**	**Do not know** ***n*** **(%)**	**Disagree** ***n*** **(%)**	
Only smokers use e-cigarettes	42 (20%)	48 (23%)	119 (57%)	29 (22%)	26 (19%)	79 (59%)	9 (9%)	15 (15%)	74 (76%)	**0.002**
E-cigarettes are safe for health	54 (26%)	51 (24%)	104 (50%)	30 (22%)	31 (23%)	73 (55%)	18 (18%)	27 (28%)	53 (54%)	0.417
Less addictive than regular cigarettes	133 (64%)	34 (16%)	42 (20%)	75 (56%)	26 (19%)	33 (25%)	65 (66%)	17 (18%)	16 (16%)	0.599
Helpful for quitting smoking	134 (64%)	32 (15%)	43 (21%)	83 (62%)	20 (15%)	31 (23%)	54 (55%)	24 (25%)	20 (20%)	0.275
Can cause lung cancer, stroke, etc.	85 (41%)	76 (36%)	48 (23%)	42 (31%)	65 (49%)	27 (20%)	20 (20%)	62 (63%)	16 (16%)	**0.049**
Should be regulated by the government	129 (62%)	32 (15%)	48 (23%)	100 (75%)	16 (12%)	18 (13%)	64 (65%)	16 (16%)	18 (19%)	**0.005**

n, the number of individuals.

%, the percentage of the total number represented by n.

p-Value was calculated using the independent samples t-test. Significance determined at p ≤ 0.05 (two-tailed). Bold values indicate statistically significant results (*p* < 0.05).

Participants also characterized individuals who use e-cigarettes differently from those who smoke traditional cigarettes. E-cigarette users were more frequently described as stylish (*n* = 85, 20%), independent (*n* = 79, 18%), and “cool” (*n* = 77, 18%). In contrast, traditional cigarette smokers were more often perceived as inconsiderate (*n* = 109, 25%) and unattractive (*n* = 92, 21%). These findings reflect distinct social perceptions and stereotypes associated with e-cigarette vs. traditional cigarette use (Table 5).

**Table 5:** Participants description of e-cigarettes and traditional cigarettes smokers.

**Participants' descriptions**	**Traditional cigarettes**			**E-cigarettes**		
	**Very much** ***n*** **(%)**	**A little** ***n*** **(%)**	**Not at all** ***n*** **(%)**	**Very much** ***n*** **(%)**	**A little** ***n*** **(%)**	**Not at all** ***n*** **(%)**
Stylish	54 (12%)	177 (40%)	210 (48%)	85 (20%)	178 (40%)	178 (40%)
Tough	88 (20%)	175 (40%)	178 (40%)	59 (13%)	179 (41%)	203 (46%)
Cool	58 (13%)	151 (34%)	232 (53%)	77 (18%)	173 (39%)	191 (43%)
Independent	77 (18%)	173 (39%)	191 (43%)	79 (18%)	171 (39%)	191 (43%)
Unattractive	92 (21%)	188 (42%)	161 (36%)	64 (15%)	156 (35%)	221 (50%)
Immature	69 (16%)	160 (36%)	212 (48%)	65 (15%)	157 (35%)	219 (49%)
Inconsiderate	109 (25%)	186 (42%)	146 (33%)	75 (17%)	173 (39%)	193 (44%)
Trashy	49 (11%)	127 (29%)	265 (60%)	59 (13%)	124 (28%)	258 (59%)

n, the number of individuals.

%, the percentage of the total number represented by n.

### 3.5 Reported adverse effects

**Figure 2:**
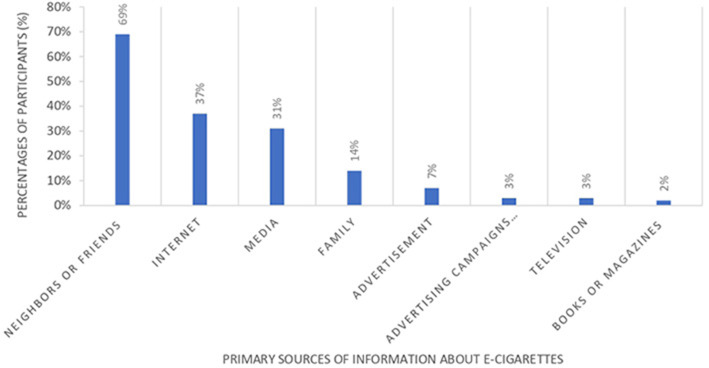
Prevalence of adverse symptoms experienced by e-cigarette users.

More than one-third of participants (*n* = 193, 44%) reported experiencing side effects associated with e-cigarette use, with the most commonly reported symptoms being shortness of breath (*n* = 78, 18%), phlegm production (*n* = 67, 15%), and chest pain (*n* = 67, 15%; Figure 2). There were no significant differences in the prevalence of symptoms among the user groups (*p* = 0.682).

## 4 Discussion

This study investigated the demographic, socioeconomic, and behavioral characteristics of e-cigarette users, ex-smokers, and dual users, alongside their perceptions and motivations for e-cigarette use in the KSA. The findings provide significant insights into the patterns of e-cigarette use and the factors influencing this behavior, contributing to the limited body of literature on this topic in the region.

The majority of participants were young males, with e-cigarette use significantly associated with younger age, male gender, and lower educational and income levels. These findings align with previous studies (13, 14), highlighting the demographic vulnerability of young adults to e-cigarette use. However, the association between lower education levels and e-cigarette use diverges from Albaik et al. (11), suggesting the need for region-specific interventions. Targeted policies, such as restricting online sales, enforcing age limits, and licensing merchants, could mitigate youth access and consumption.

Half of the participants reported daily e-cigarette use, and a notable preference for high nicotine concentrations (≥16 mg) was observed. These results contrast with the findings of Karbouji et al. (10), who reported lower levels of daily use and less frequent preference of high nicotine concentrations among smokers surveyed across Saudi regions, suggesting a potential shift toward more frequent and higher nicotine use in recent years. The widespread use of high-nicotine e-cigarettes underscores the potential for nicotine dependency, necessitating regulation by health authorities. The Saudi Ministry of Health should consider setting limits on nicotine content in e-cigarette products to promote harm reduction and support cessation efforts. In addition to regulating the nicotine concentration in e-cigarettes, increasing taxation on these products could be an effective tool to reduce consumption, particularly among younger age groups with lower disposable income. Assessing the impact of existing and potential taxes on usage patterns is essential to inform public health policies better. A recent study by Diaz et al. (15) has demonstrated the association between e-cigarette excise tax increases and reductions in youth use, highlighting the importance of fiscal policy measures in controlling e-cigarette consumption.

Saudi Arabia has already implemented significant tobacco control measures, including a 100% excise tax on tobacco products, sales restrictions to minors, and bans on smoking in public places (8, 9). These efforts provide a regulatory foundation that could be expanded to include specific e-cigarette policies. However, potential barriers to policy adoption include enforcement challenges, industry influence, and varying levels of public awareness about e-cigarette risks. Facilitators may consist of the high level of public support for regulation observed in this study (61%) and alignment with existing public health priorities. Lessons from countries such as the UK (16), where nicotine content is limited to 20 mg/mL, and Australia (17), where e-cigarettes containing nicotine require a prescription, demonstrate that strict regulatory frameworks can be effectively implemented. Future policies in Saudi Arabia could benefit from adopting a systematic evaluation framework, such as monitoring the impact of tax policies or sales restrictions on youth usage rates, to ensure effectiveness and guide adjustments over time.

The primary motivations for e-cigarette use were smoking cessation, enjoyment, and perceived safety compared to traditional cigarettes. These findings are consistent with those of AlHumaidan et al. (6) in Riyadh, where similar reasons—particularly smoking cessation and curiosity—were cited, highlighting common motivational patterns among young adults in different regions. Retailers and online stores were the primary sources of e-cigarettes, echoing patterns reported by Puteh et al. (13). This highlights the need for stricter regulations on e-cigarette sales and distribution channels to reduce accessibility.

Participants frequently reported adverse effects such as shortness of breath, phlegm production, and chest pain, which aligns with the side effects documented in both Karbouji et al. (10) and Albaik et al. (11), confirming that these symptoms are consistently observed across various populations in Saudi Arabia. Despite these concerns, many participants viewed e-cigarettes as less harmful and addictive compared to traditional cigarettes, consistent with findings from Zhu et al. (18) and Tan et al. (19). These perceptions may contribute to their popularity, underscoring the importance of public health campaigns to correct misconceptions and educate the public on potential risks.

A majority of participants advocated for government regulation of e-cigarettes, including restrictions on sales to minors and unregulated outlets. These findings align with Karbouji et al. (10) and reinforce the need for comprehensive policy measures. Public health initiatives should focus on raising awareness about the potential harms of e-cigarettes and promoting evidence-based cessation strategies. The correlation analysis provided insight into potential relationships between behavioral patterns and intentions regarding e-cigarette use. The weak but significant positive correlation between frequency of use and type of e-juice suggests that individuals who use e-cigarettes more frequently may prefer certain types of e-juice, reflecting a greater level of engagement with or dependence on the product. The moderate negative correlation between frequency of use and intention to be a regular smoker in the following year is noteworthy, as it indicates that those who currently use e-cigarettes more frequently are less likely to express an intention to continue regular use, which could reflect ambivalence, experimentation, or ongoing efforts to quit. The multinomial logistic regression further highlighted how demographic and socioeconomic factors shape patterns of use. Specifically, gender, region, marital status, and education were significant predictors of smoking group membership, underscoring the influence of these variables on both e-cigarette uptake and dual use. These findings align with prior research demonstrating that complex interactions between personal, social, and contextual factors shape e-cigarette use patterns (6). They also highlight the need for tailored interventions that consider these demographic and regional differences when designing public health strategies to address e-cigarette use.

In addition to national public health policies led by the Ministry of Health, there is a clear need to involve the Ministry of Education in prevention efforts to address the growing use of e-cigarettes among young people. Schools and universities provide important platforms for early intervention, given that a large proportion of e-cigarette users are young adults, as demonstrated in this study and others. Integrating evidence-based tobacco and e-cigarette prevention content into health education curricula, alongside targeted awareness campaigns and peer-led initiatives, could help correct misconceptions about e-cigarette safety and reduce uptake. Furthermore, university programs could offer cessation support services for students already using e-cigarettes. Coordinated efforts between the Ministry of Health and the Ministry of Education would contribute to a more comprehensive and sustainable national strategy to reduce e-cigarette use among youth in Saudi Arabia.

In addition to regulatory and educational interventions, it is important to recognize the influence of sociocultural factors on e-cigarette use in Saudi Arabia. The Kingdom's unique social norms, gender dynamics, and family structures may shape both motivations for use and perceptions of risk. For example, the predominance of male participants and higher reported use among males likely reflects underlying gender-related norms regarding tobacco and nicotine use, where smoking behaviors are more socially tolerated or concealed differently across genders (6, 10). Furthermore, the strong influence of family and peers—as reflected in our finding that 90% of participants lived with or had close contact with another e-cigarette user—highlights the role of interpersonal relationships in shaping health behaviors (3). Cultural perceptions of e-cigarette users as more “stylish” or “independent” may also reflect broader social narratives that contribute to the normalization of e-cigarette use among youth (3, 6). These sociocultural factors need to be explored more in future studies using interviews or mixed research methods. This will help us better understand how they affect e-cigarette use and guide the design of interventions that fit the local culture.

### 4.1 Strengths, limitations, and future research

This study provides valuable insights into e-cigarette use in the KSA but has some limitations. Self-reported data may be subject to recall or social desirability bias. Additionally, the survey was administered in English only, which may have limited participation or introduced selection bias, as individuals with lower English proficiency may have been less likely to participate despite being part of the target population. Future studies should consider offering the survey in both Arabic and English to enhance inclusivity and generalizability. Also, as most participants in the current study were young males, the findings may not fully capture the views and behaviors of older adults or females in Saudi Arabia. Future studies should aim for more balanced and representative samples to enhance generalizability. Further research is needed to explore the long-term health effects of e-cigarette use and to assess the impact of regulatory interventions on usage patterns. Our findings highlight the need for future research to explore gender-specific patterns in e-cigarette use, particularly given the evolving social perceptions of vaping in the region. Such research could provide valuable insights into whether shifting attitudes toward e-cigarettes influence vaping behaviors differently among males and females, and help inform the development of gender-sensitive public health interventions. An additional limitation of our study is that we did not conduct a subgroup analysis comparing reported adverse symptoms between users of nicotine-free and nicotine-containing e-cigarettes. Future research should explore this relationship to understand better the potential role of nicotine in contributing to specific health effects, such as chest pain. An area that warrants future research is the relationship between the length of daily e-cigarette use and the preference for higher nicotine concentrations, as this could be important for understanding nicotine dependence patterns and guiding more targeted health interventions. Another limitation of our study is that it did not explore in depth the sociocultural context that influences e-cigarette use in Saudi Arabia. Although our findings touch on aspects of peer and family influence and social perceptions, the study design did not allow for a detailed examination of how cultural, religious, and gender-related factors shape behaviors and attitudes. Future research should incorporate qualitative or mixed-method approaches to capture these important contextual factors, which could provide richer insights into the drivers of e-cigarette use and support the development of tailored public health interventions. A further limitation of this study is that the market availability and prevalence of different types of e-cigarettes and nicotine concentrations in Saudi Arabia were not investigated. It is recommended that future studies include market surveys or be conducted in collaboration with regulatory bodies to better understand the types of products accessible to consumers. In this way, user preferences and knowledge gaps could be better explained, and stronger regulatory policies could be informed.

## 5 Conclusions

E-cigarette use is common among younger males in KSA, influenced by demographic and socioeconomic factors, with motivations including smoking cessation, pleasure, and cost. Despite being viewed as safer alternatives to traditional cigarettes, adverse effects like shortness of breath and chest pain were frequently reported. Comprehensive public health policies, including education programs, sales restrictions, and nicotine regulation, are urgently needed to address the rising prevalence and risks of e-cigarettes. Continuous monitoring and further research are essential to assess trends, risk factors, and the relationship with tobacco use to inform effective strategies.

## Data Availability

The raw data supporting the conclusions of this article will be made available by the authors, without undue reservation.
